# Systemic and sex-biased regulation of OBP expression under semiochemical stimuli

**DOI:** 10.1038/s41598-018-24297-z

**Published:** 2018-04-16

**Authors:** Débora Pires Paula, Roberto Coiti Togawa, Marcos Mota do Carmo Costa, Priscila Grynberg, Natália Florêncio Martins, David Alan Andow

**Affiliations:** 1 Embrapa Genetic Resources and Biotechnology, Parque Estação Biológica, W5 Norte, P.O. Box 02372, Brasília, DF 70770-917 Brazil; 2 Department of Entomology, University of Minnesota, 219 Hodson Hall, 1980 Folwell Ave., St. Paul, MN 55108 USA

## Abstract

Constitutive expression of Odorant-Binding Proteins (OBPs) in antennae and other body parts has been examined mainly to infer their involvement in insect olfaction, while their regulation in response to semiochemical stimuli has remained poorly known. Previous studies of semiochemical response were basically done using electrophysiology, which integrates the response of the set of OBPs present in an antenna or sensillum, without revealing the regulation of OBPs or which ones might be involved. In this study we used boll weevil as a model and mined its OBPs by RNA-Seq to study their simultaneous antennal expression by qPCR under controlled semiochemical stimuli with aggregation pheromone and plant volatiles. In the absence of a semiochemical stimulus, 23 of 24 OBPs were constitutively expressed in the antenna in both sexes. Semiochemicals changed systemically the expression of OBPs in both sexes. There were different patterns of up- and down-regulation in female antennae for each semiochemical stimulus, consistent with female chemical ecology. On the other hand, the only response in males was down-regulation of some OBPs. We suggest that these systemic changes in OBP expression might be related to enhancing detection of the semiochemical stimuli and/or priming the olfactory system to detect other environmental chemicals.

## Introduction

Odorant-Binding Proteins (OBPs) play a key role in the first step of insect olfaction. They act as a semiochemical solubilizer, transporter and ligand filter, and mediate the activation of the Olfactory Receptors (ORs) to enable insect olfactory perception^1^. Considerable *in vitro* research in many insect species has documented that many OBPs have a binding preference for or higher affinity to specific compounds^2–4^, or have considerable flexibility to bind to a broad range of compounds [*e.g*.^5–9^].

Recent research has documented the constitutive expression of OBPs in insects [*e.g*.^10,11^], and for the vast majority of OBPs, constitutive expression levels are higher in antennae than in other body parts, leading most authors to conclude that the OBPs are involved in olfaction of semiochemicals. However, the regulation of expression of OBPs in response to environmental semiochemicals, remains poorly known. Does the presence of semiochemical affect the level of expression of OBPs? Does exposure to a semiochemical trigger systemic up-regulation and/or down-regulation of a set of OBPs? If there is systemic up- and/or down-regulation, does it enhance the detection of the semiochemical or does it prime the olfactory system to detect other environmental chemicals?

We use the boll weevil, *Anthonomus grandis* Boheman (Coleoptera: Curculionidae), as a model to address these questions, because this species has a known chemical ecology. Boll weevil is one of the major pests of cotton in the Americas^12^. In the late 19th century it spread from Mexico to the USA and to Central and South America^13^ causing substantial economic losses in cotton production, and while it has been eradicated from large parts of the USA, it remains a key pest wherever it still occurs.

Males locate the host plant through volatiles emanating from the host plant around the time of flower bud formation^14,15^. Several cotton volatiles have been identified as attractants or stimulants to virgin male boll weevils^16–34^. After feeding on cotton for 3–5 days^35^, male weevils release a pheromone highly attractive to virgin females^14–16^, and somewhat attractive to males and mated females^36,37^. Thereby, female weevils can find mates and host plants, and later oviposit in the cotton fruiting structures to initiate reproduction. In addition to attraction, the neurophysiological response to several of these semiochemicals has been extensively studied, showing that boll weevil uses different antennal neurons and sensillae for pheromones and plant volatiles, and both sexes are sensitive to stimulation by both semiochemicals^20^.

To verify the systemic regulation of OBP expression in *A. grandis* and consider if enhanced detection or priming is occurring, in this work we identified putative OBP transcripts through RNA-Seq and studied their expression response against two semiochemical stimuli: aggregation pheromone and plant volatiles. Initially, as a reference, we examined their constitutive expression in antennae and legs in the absence of semiochemical stimulation. Then, we stimulated virgin adults of each sex with either pheromone or a mixture of plant volatiles and analyzed the expression of the OBP transcripts. We observed that the same set of OBPs was constitutively expressed in both sexes, and a systemic regulation of AgraOBP expression happened in response to semiochemical stimuli, with a sex-biased outcome.

## Methods

### Boll weevil OBP mining

We mined the boll weevil OBP transcripts through transcriptome data deposited by Firmino *et al*.^38^ at GenBank (20,246 sequences, accession code PRJNA177772). To assess the completeness of the transcriptome assembly we used CEGMA (Core Eukaryotic Genes Mapping Approach)^39^. The transcripts (contigs) were annotated using BLAST2GO v. 2.8.0^40^. The initial search of the National Center for Biotechnology Information (NCBI) nonredundant (nr) protein database was conducted with the BLASTx algorithm with an E-value less than 10e-5. In addition, insect protein sequences were retrieved from the NCBI database and searched against *A. grandis* transcript sequences using the BLASTx algorithm (E-value 10e-30). InterproScan was applied to candidate sequences. Only those with conserved protein domains associated with olfactory proteins were retained for further analysis; these were Pheromone/General Odorant Binding Protein (IPR006170), and Insect Odorant-Binding Protein A10/Ejaculatory Bulb-Specific Protein 3 (IPR005055). The putative contigs related to OBPs were screened for open reading frames (ORFs) using the software ORF FINDER (http://www.ncbi.nlm.nih.gov/gorf/gorf.html) to identify the coding regions. We performed *in silico* characterization of the full-length putative OBPs to confirm their annotation (Supplementary Information 1) and we have named them by the first letter of the genus followed by the first three letters of the species name (*e.g*., AgraOBP) followed by a number in increasing scaffold order. They were deposited at GenBank using the submission tool BankIt.

### Semiochemical assays

Insects for the semiochemical studies were reared according to Monnerat *et al*.^41^. Adult beetles were harvested from the rearing containers within 1 h of emergence and placed individually in 1.5 mL microtubes. Beetles were sexed using the method of Sappington and Spurgeon^42^ and separated by sex in 30 × 30 cm plastic boxes with water, 1 mL of artificial diet^43^ and a fresh cotton boll and leaves. Each sex was isolated in a separate rearing chamber at 13 h photophase, 25 ± 4 °C and 60 ± 10% RH until the bioassay. All food sources were removed on the third day, and beetles were maintained on water until they were used in the experiments when they were five days old, at which time they would be maximally responsive to semiochemicals^44,45^. At least 2 h before the experiment, beetles were isolated by sex and placed individually into 1.5 mL microtubes containing a small hole in the bottom and top to allow air flux during the semiochemical experiments and isolated by sex until the experiment.

Chemical sources of the semiochemicals are described in Supplementary Information 1. Total pheromone and each plant volatile compound was mixed to a concentration of 4% v/v. Individual boll weevils were exposed to each semiochemical stimulus by pushing charcoal-filtered, humidified air at 0.2 L.min^−1^ through a Pasteur pipette containing the stimulus and into the microtube containing a beetle. The air handling system is described in detail in Magalhães *et al*.^46^. The microtube had a small hole in the lid that fit the pipette tip and a small hole in the bottom to exhaust the air. Semiochemical stimuli were produced by pipetting 25 μL of one of the solutions onto an 8 × 18 mm piece of filter paper, allowing it to air-dry for 1 min, and loading into a Pasteur pipette; 25 μL of *n*-hexane was used as a negative control. This is equivalent to a 1 μg dose of total pheromone and each plant volatile compound. Dickens (1984) showed that this dose was above the detection threshold and below the saturation dose for boll weevil based on electrophysiology. The semiochemical stimuli were replaced if they were older than 15 min. There was a total of six treatments: two sexes x three semiochemical stimuli (pheromone, plant volatiles -PVOC- and control with *n*-hexane), with 49–51 beetles each.

As females do not give off any attractive semiochemicals^47^, they were tested before males. Each individual was exposed to the semiochemical stimulus for 15 s and immediately killed by immersion in liquid nitrogen and stored at −80 °C until further processing.

### OBP expression analysis

Antennae and legs of the males and females were dissected using RNase-free instruments and were immediately immersed in 1 mL of TRIzol (Invitrogen), according to the semiochemical stimulation and sex. They were macerated and homogenized using a different RNase-free mortar and pestle for each sample, and RNA was extracted using a PureLink RNA Mini kit (Ambion by LifeTechnologies), according to manufacturer instructions. DNase treatment and purification was processed on-column using a PureLink DNase provided by a complementary kit from Ambion. The RNA yield and quality was analyzed by nanodrop. For cDNA synthesis, we used SuperScript III First-Strand Synthesis System for qPCR (Invitrogen by LifeTechnologies) with 1 μg of total RNA, according to the manufacturer’s instructions. Primer pairs were designed (see Table S1 in Supplementary Information 2) based on the putative transcripts to determine transcript abundance of the OBPs in these treatments using qPCR. The qPCR reactions (12.5 μL) were prepared using Thermo Scientific Maxima SYBR Green/ROX qPCR Master Mix (2X), with 500 ng of cDNA and each specific primer pair at 0.3 μM. The amplifications were performed in 384-well plates with a Roche Applied Science LightCycler® 480 Real-Time PCR System using a two-step cycling protocol (initial denaturation at 95 °C for 10 min, and 40 cycles of denaturation at 95 °C for 15 s and annealing/extension at 60 °C for 60 s).

Each qPCR reaction for each sample was performed in at least three technical replicates. No-template controls (NTC) were included for every primer pair in each experiment; no amplification of the target amplicons was observed in the NTCs. Melting curve analysis was performed to test locus-specificity of reaction products. Theoretical melting peaks were determined using μMelt^SM^^48^ with Unified-Santa Lucia thermodynamic set and default parameters. Wells with peaks >3°C different from the theoretical peak or with multiple peaks were considered non-quantifiable.

The starting concentration per sample (N_0_) was estimated from the raw fluorescence data (R_n_) using the software LinReg version 2016.2^49–51^, for each plate and each amplicon on each plate separately, excluding wells without amplification, requiring a strictly continuous log-linear phase, and eliminating plate effects^52^. Subsequent statistical analysis used N_0_ because of variation in the empirically estimated PCR efficiencies. Because the qPCR samples from the different bioassays had slightly different numbers of individual adults in them (*n* = 49 to 51 beetles), the N_0_ values were normalized by the number of individuals to enable comparison across bioassays.

We used EF1 and GAPDH as reference genes. We calculated a weighted mean and standard error of the log_10_ N_0_ for each gene and each treatment using inverse variance as the weight. We used the means and standard errors to adjust the log_10_ N_0_ and standard errors for all treatments and amplicons using the formula for the variance of the product of two independent random variables.

### Statistical analysis

Two statistical analyses were conducted. The first focused on expression in the controls, to understand which transcripts are constitutively expressed in each sex and either antennae or legs. Expression was compared in the antennae and legs for each AgraOBP and sex by ANOVA using Proc GLM in PC-SAS 9.4^53^. The second analysis focused on the effect of semiochemical exposure on the expression of the AgraOBP transcripts in antennae. We determined which genes were turned on, turned off, up-regulated or down-regulated relative to the controls. For each AgraOBP, the level of gene expression was analyzed by ANOVA, with Log_10_N_0,*ik*_ = μ + *s*_*i*_ +  + *t*_*k(i)*_ + ε_*ik*_, where μ is the grand mean, *s*_*i*_ is the effect of sex *i*, *t*_*k(i)*_ is treatment within *s*, and ε_*ik*_ is the error. These analyses were conducted using Proc GLM in SAS 9.4^53^. The effect of the semiochemicals was quantified and tested with the Estimate statement with unequal sample sizes. Under the null hypothesis of no significant effects, the *p*-values of the tests will be uniformly distributed between 0 and 1. The histogram of the *p*-values for the semiochemical statistical tests indicated that *p*-values less than 0.01 are biologically significant. Statistical comparisons of the number of OBPs responding to stimuli between females and males or pheromone and plant volatiles were done using contingency table analysis with a log-linear model and binomial error, and quantitative comparisons of up- and down-regulation were done with a two-sample *t*-test.

### Data availability

The datasets generated during and/or analyzed during the current study are available from the corresponding author on reasonable request.

## Results and Discussion

### OBP transcripts in *Anthonomus grandis*

For the boll weevil transcriptome with completeness of 67.74% (Table S2), we identified 23 OBPs and one GOBP (an OBP subcategory) (Table 1), a number greater than the average found in Coleoptera (17 ± 15, *n* = 46, Table S3). Most of the AgraOBPs were identified full-length, similar to reports for other coleopterans^54–61^.

**Table 1:** Odorant-Binding Protein transcripts identified in *Anthonomus grandis*.

Gene name	Search tool	Accession number	Completeness	Best Blastx hit					
				Protein	Species	Query coverage (%)	Identity (%)	E-value	Accession number
AgraOBP1	Blast2GO	MF186622	Missing 5′ and 3′	OBP	*Rhynchophorus ferrugineus*	100	49	6e-31	AMK48596.1
AgraOBP2	Blast2GO	KY826455	full-length	OBP2	*Dendroctonus ponderosae*	80	52	5e-58	AGI05158.1
AgraOBP3	Blast2GO	KY826456	full-length	OBP9	*Dendroctonus ponderosae*	97	47	4e-37	AKK25135.1
AgraOBP4	Blast2GO	KY826457	full-length	OBP4	*Dendroctonus ponderosae*	97	40	1e-44	AGI05167.1
AgraOBP5	Blast2GO	KY826458	full-length	OBP	*Lissorhoptrus oryzophilus*	84	77	5e-67	AHE13793.1
AgraOBP6	Blast2GO	KY826459	full-length	OBP2	*Dendroctonus ponderosae*	96	71	2e-66	AIY61045.1
AgraOBP7	Blast2GO	KY826460	full-length	OBP13	*Dendroctonus armandi*	78	33	3e-16	ALM64971.1
AgraOBP8	Blast2GO	KY826461	full-length	OBP8	*Dendroctonus ponderosae*	92	50	7e-40	AGI05175.1
AgraOBP9	Blast2GO	KY826462	full-length	OBP20	*Dendroctonus ponderosae*	81	46	4e-31	AKK25144.1
AgraOBP10	Blast2GO	KY826463	full-length	PBP11	*Cyrtotrachelus buqueti*	68	43	2e-23	APG79372.1
AgraOBP11	Blast2GO	MF186631	Missing 5′	OBP	*Lissorhoptrus oryzophilus*	86	61	2e-31	AHE13799.1
AgraOBP12	Blast2GO	KY826464	full-length	OBP6	*Rhynchophorus ferrugineus*	84	37	7e-17	ANE37550.1
AgraOBP13	Blast2GO	KY826465	full-length	OBP13	*Dendroctonus armandi*	95	35	1e-20	ALM64971.1
AgraOBP14	Blast2GO	KY826466	full-length	PBP3	*Cyrtotrachelus buqueti*	81	37	9e-21	APG79364.1
AgraOBP15	Blast2GO	KY826467	full-length	OBP13	*Dendroctonus armandi*	86	30	8e-11	ALM64971.1
AgraOBP16	Blast2GO	KY826468	full-length	OBP19	*Dendroctonus ponderosae*	87	62	1e-44	AKK25143.1
AgraOBP17	InterPro	MF186632	Missing 5′ and 3′	PBP7	*Cyrtotrachelus buqueti*	80	42	2e-12	APG79368.1
AgraOBP18	InterPro	KY826469	full-length	OBP6	*Dendroctonus ponderosae*	73	37	6e-09	AKK25134.1
AgraOBP19	InterPro	KY826470	full-length	OBP8	*Dastarcus helophoroides*	61	24	0.002	AIX97054.1
AgraOBP20	InterPro	MF186633	Missing 5′ and 3′	PBP17	*Cyrtotrachelus buqueti*	72	49	7e-08	APG79378.1
AgraOBP21	InterPro	KY826471	full-length	OBP	*Lissorhoptrus oryzophilus*	91	29	3e-06	AHE13798.1
AgraOBP22	InterPro	MF186634	Missing 5′	OBP5	*Dendroctonus armandi*	97	35	0.003	ALM64967.1
AgraOBP23	InterPro	KY826472	full-length	OBP6	*Dastarcus helophoroides*	82	41	3e-26	AIX97052.1
AgraGOBP1	InterPro	MF186635	Missing 5′ and 3′	GOBP70	*Aethina tumida*	100	49	2e-16	XP_019871740.1

Several pieces of information together support the annotation of these mined AgraOBPs. They have similar physicochemical properties as other OBPs identified in Coleoptera (Table 2). Typically OBPs have been found with 135–220 amino acids (aa), acid pI (4.5–5.5) and a signal peptide of 16–20 residues at the amino-terminal end^24,25^. Some AgraOBPs were smaller (*e.g*., AgraOBP15 was the smallest with 105 aa), with basic pI (AgraOBP9 = 8.88), or without the predicted signal peptide (AgraOBP2 and AgraOBP4). Others reported coleopteran OBPs that were smaller than typical^54,62–69^, have a basic pI^66,68,70–72^, or lack the signal peptide^56,57,59,62,66,67^.

**Table 2:** Physicochemical predictions for the putative full-length AgraOBPs obtained from boll weevil.

ID	AA	pI	SignalP	Conserved Cys	AA position of the InterPro domain
AgraOBP2	215	6.54	No	Atypical	70–142^a^; 77–153^b^
AgraOBP3	133	6.88	1–17	Minus-C	19–127^a^; 16–125^b^
AgraOBP4	191	5.51	No	Minus-C	1–65 and 80–181^a^; 1–63 and 76–181^b^
AgraOBP5	149	5.15	1–19	6	201–129^a^; 20–127^b^
AgraOBP6	135	4.58	1–18	6	22–133^a^; 24–126^b^
AgraOBP7	142	5.41	1–16	Minus-C	22–129^a^; 20–129^b^
AgraOBP8	146	4.65	1–21	6	6–140^a^; 21–133^b^
AgraOBP9	139	8.88	1–19	Minus-C	30–135^a^; 24–134^b^
AgraOBP10	162	6.19	1–16	Minus-C	32–136^a^; 29–135^b^
AgraOBP12	118	4.84	1–16	Minus-C	27–117^a^; 21–106^b^
AgraOBP13	136	5.63	1–18	Minus-C	22–131^a^; 20–130^b^
AgraOBP14	164	4.79	1–21	Minus-C	29–135^a^; 29–132^b^
AgraOBP15	105	6.82	1–16	Minus-C	8–95^a^; 20–98^b^
AgraOBP16	134	5.04	1–19	Minus-C	19–130^a^; 20–126^b^
AgraOBP18	133	5.29	1–19	Minus-C	55–123^a^
AgraOBP19	141	4.83	1–23	6	39–135^a^; 42–135^b^
AgraOBP21	127	5.11	1–17	Minus-C	24–122^a^; 21–122^b^
AgraOBP23	138	6.08	1–16	Minus-C	23–135^a^; 20–124^b^

AA: Amino acid; pI: Isoelectric point; Cys: cysteine; SignalP: signal peptide amino acid location. Six “Conserved Cys” is classical OBP. InterPro domains: IPR06170/PBP/GOBP (^a^SSF47565 Insect pheromone/odorant-binding proteins; ^b^PFAM PF01395 PBP_GOBP).

Nearly all the putative AgraOBPs had the predicted domain of a Pheromone/General Odorant-Binding Protein (IPR006170) and all had the expected predicted tertiary structures for OBPs with globular configurations of five-six alpha helices surrounding a hydrophobic binding pocket (Fig. S2 in Supporting Information 2). The hydrophobic binding pocket was lined with several hydrophobic amino acids, such as branched chain amino acids (Ile, Leu, Val) and aromatic residues (Tyr, Phe), and the alpha-helices were held together by three disulphide bridges between Cys1–Cys3, Cys2–Cys5 and Cys4–Cys6, as expected for an OBP structure^1^.

**Figure 1:**
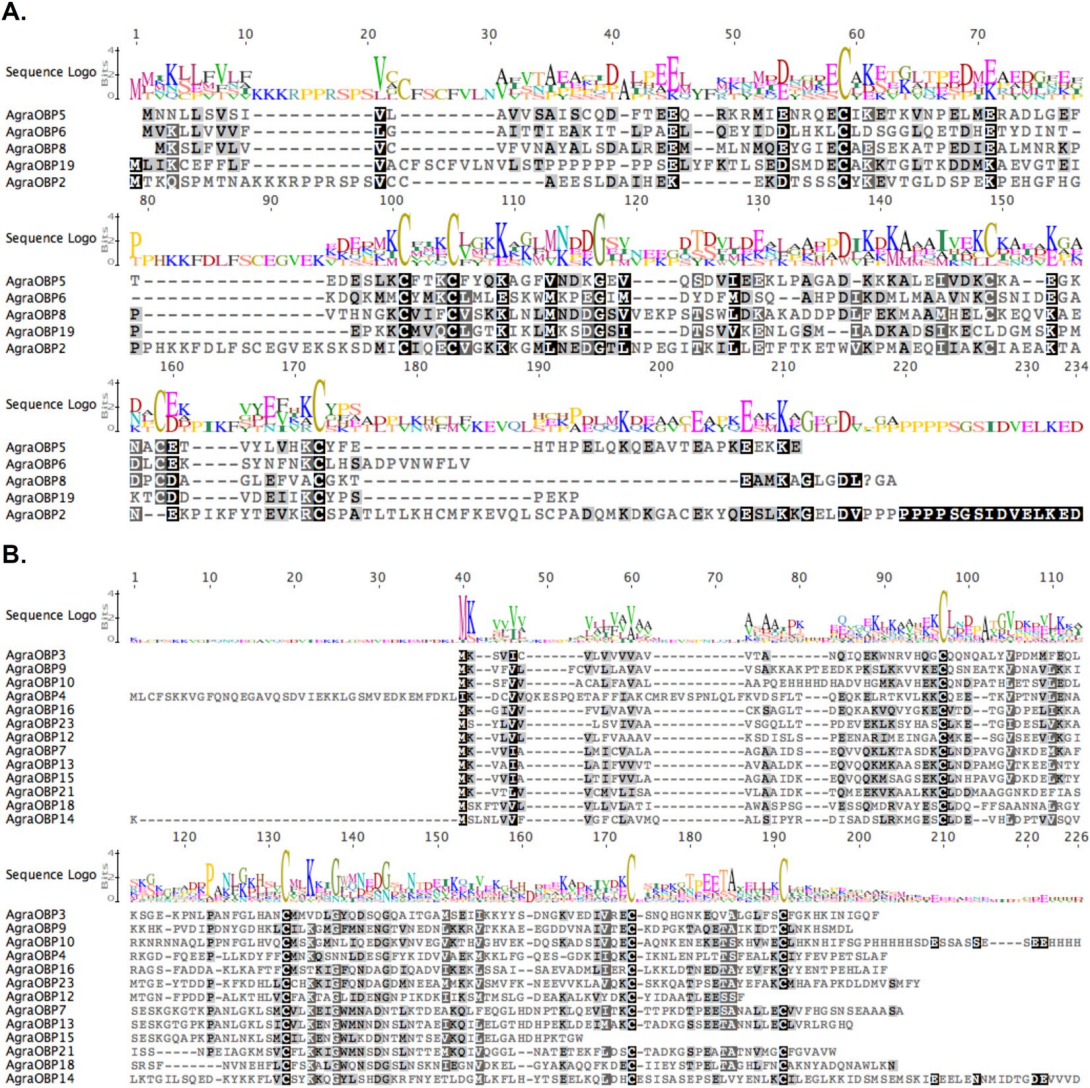
Alignment of the deduced amino acid sequences identified in the transcriptome of *Anthonomus grandis*. (**A**) Classical and atypical OBPs; (**B**) Minus-C OBPs. Similarity is scored by matrix Blosum62 where the black color indicates 100% similarity, darker grey 100% > similarity ≥ 80%, lighter grey 80% > similarity ≥ 60% and white color similarity < 60%. The sequence logo is at the top of the alignment. The conserved Cys are indicated by sequence logo.

Regarding the Cys-pattern, we found in *A. grandis* four classical OBPs, 13 Minus-C, and one atypical OBP (Fig. 1). All putative classical AgraOBPs had the strictly conserved three residues between C2–C3 and had the Cys motif-pattern of OBPs expected for Coleoptera (C1-X_23–44_-C2-X_3_-C3-X_36–43_-C4-X_8–12_-C5-X_8_-C6) (X denotes any amino acid)^73^. All of the Minus-C AgraOBPs had the four conserved Cys and lacked the C2 and C5 conserved Cys, as expected^74^, and had the same pattern X33-C1-X30-C2-X39-C3-X16-C4-X12, except for AgraOBP12 (lacked only the fourth Cys) and AgraOBP15 (lacked the third and the fourth Cys). The atypical OBP resembled two fused OBPs with predicted size of approximately 24 kDa, has additional conserved cysteines in an extended C-terminal region^75^, but lacked the fifth conserved Cys and has no signal-peptide in the N-terminal end. Other coleopterans also were observed with most of the OBPs assigned as Minus-C^55,56,61,66,72,76^. The Plus-C pattern was also not identified in the other coleopterans^60–62,72^.

### Constitutive expression of OBPs in antennae and legs

**Figure 2:**
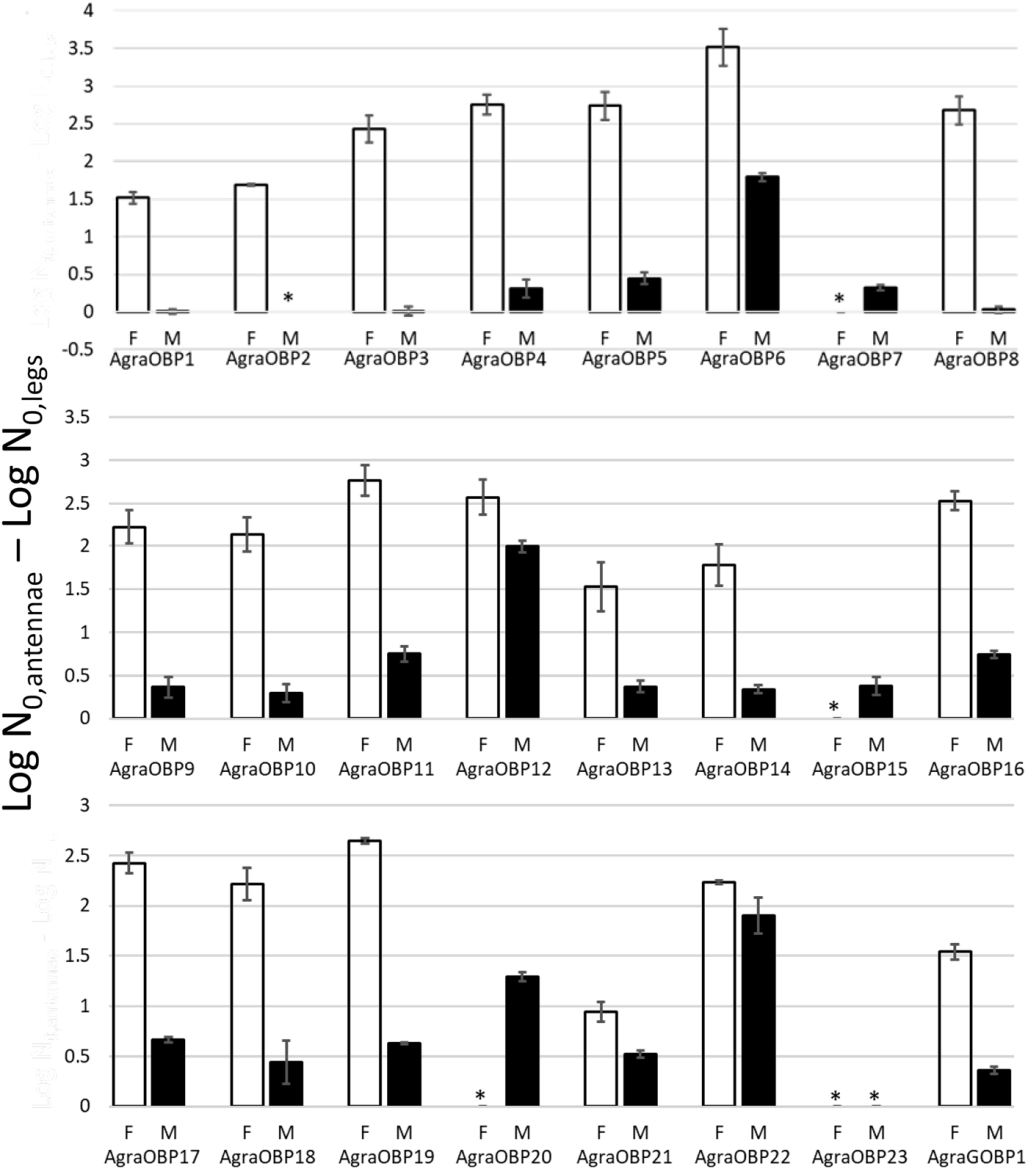
Expression of AgraOBP transcripts in antennae compared to legs in female (F) and male (M) control boll weevils. Expression is the fold-increase in log_10_ relative fluorescence units (N_0_) obtained by qPCR, with standard error bars. * are effects where there was no expression in legs or both antennae and legs.

All of the putative AgraOBP transcripts were amplified in the qPCR assays, verifying their existence, and all 24 were constitutively expressed in female and male antennae and legs, except two cases. AgraOBP23 was constitutively expressed only in male antennae, and AgraOBP20 was not expressed in female legs. For all others, transcripts were expressed at higher levels in antennae than in legs, and significantly higher in 77% of the AgraOBPs for females and 43% of the AgraOBPs for males (Fig. 2). The constitutive expression of many OBP transcripts has been reported in other Coleoptera and expression was predominantly found in the antenna and was sex-biased^54,56,62,66,70,77,78^ except in *Phyllotreta striolata*^60^. Higher expression in antennae versus other body parts indicates that the OBPs are involved in olfaction^8^, thus, all of the AgraOBPs could be involved in antennal olfaction.

### OBP expression in response to pheromone and plant volatiles

**Figure 3:**
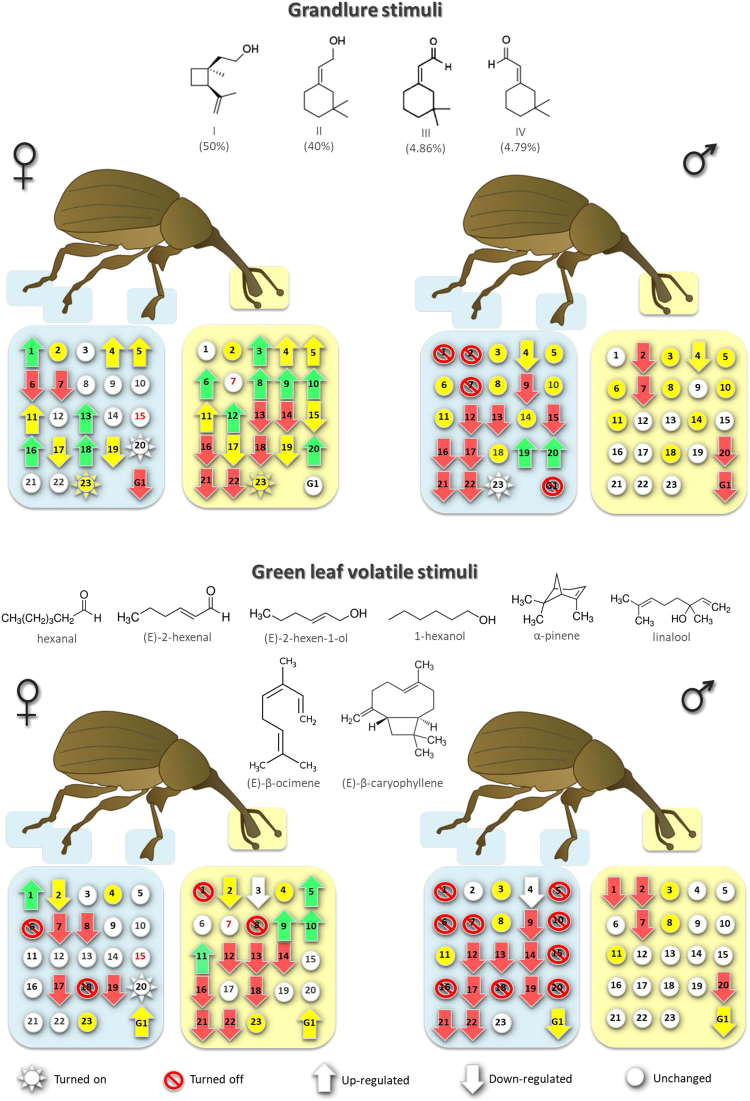
Response of AgraOBP transcripts expression to semiochemical stimuli. Plant leaf volatile compounds were combined in *n*-hexane (1 μL each/25 μL solution). G1: GOBP1. Each number inside the symbols corresponds to an AgraOBP type. Responses are relative to controls. Red numbers means not quantifiable expression. All changes are significant at *p* < 0.01. Boll weevil illustration was created by Tracey Saxby (IAN/UMCES, http://ian.umces.edu/imagelibrary/displayimage-4270.html).

The semiochemicals altered the expression of the AgraOBP transcripts in adult antennae (Figs 3 and S3 in Supplemental Information 2). Females up-regulated 10 AgraOBPs and turned on one in response to pheromone, and turned on five in response to plant volatiles. Seven and one of these were up-regulated or turned on only in response to pheromone or plant volatiles (four were up-regulated by both stimuli), respectively. The seven that were uniquely up-regulated by pheromone in female antennae were AgraOBP3 (550-fold), AgraOBP4 (53,000-fold), AgraOBP6 (150-fold), AgraOBP8 (190-fold), AgraOBP12 (310-fold), AgraOBP20 (390-fold), and AgraOBP23 (turned on). This suggests that there is redundancy in ligand-specificity for the four pheromone compounds and/or that other non-pheromone specific OBPs were up-regulated. Only AgraGOBP1 was uniquely up-regulated by the mixture of 8 plant volatiles, and it could be that it has ligand-specificity for at least one of the compounds. Males did not up-regulate any AgraOBP when exposed to either semiochemical stimulus. Expression of most male AgraOBP transcripts remained unchanged except five that were down-regulated when exposed to pheromone (AgraOBP2, AgraOBP4, AgraOBP7, AgraOBP20 and AgraGOBP1) or plant volatiles (AgraOBP1 and again for AgraOBP2, AgraOBP7, AgraOBP20 and AgraGOBP1). In male antennae, only AgraOBP1 and only AgraOBP4 were uniquely unchanged in response to pheromone and plant volatiles, respectively.

Pheromone turned on the non-expressing antennal AgraOBPs and did not turn off any AgraOBPs. AgraOBPs were up-regulated or turned on in females but not in males (Fig. 3, 10 female, 0 male, *g*^2^ = 15.07, 1 *df*, *p* = 0.0001). Quantitatively, up-regulated AgraOBPs in females increased expression a median of 345-fold (*n* = 10) (Fig. S3 in Supporting Information 2). About twice as many transcripts were down-regulated in females than in males (9 versus 5), but this was not significantly different (*g*^2^ = 1.66, 1 *df*, *p* = 0.1976). The median down-regulation in females (94-fold, *n* = 9) was not different from males (median 175-fold, *n* = 5, *t*-test, *p* = 0.7163). Only 13% of the AgraOBP transcripts in female antennae did not change the expression level when exposed to pheromone, while in male antennae 78% did not change (*g*^2^ = 21.52, 1 *df*, *p* = 3.49 × 10^−6^).

The plant volatiles did not turn on AgraOBP23 in female antennae as the pheromone did, and turned off AgraOBP1 and AgraOBP8. Again, only females up-regulated AgraOBPs (Fig. 3, 5 female, 0 male, *g*^2^ = 5.00, 1 *df*, *p* = 0.0252). Quantitatively, up-regulated AgraOBPs in females increased expression a median of 1470-fold (*n* = 5) (Fig. S3 in Supporting Information 2). Although more transcripts were down-regulated or turned off in females than in males (9 versus 5), this was not significantly different (*g*^2^ = 1.91, 1 *df*, *p* = 0.1659). For the AgraOBPs that were down-regulated, the median down-regulation in females (150-fold, *n* = 10) was less than that in males (median 920-fold, *n* = 5, *t*-test, *p* = 0.0489). Fewer (30%) of the AgraOBP transcripts were not affected by plant volatiles in females than in males (78%, *g*^2^ = 11.07, 1 *df*, *p* = 0.0009).

Several AgraOBPs had similar responses to both semiochemical stimuli (Figs 3 and S3 in Supporting Information 2). One group was AgraOBP5, AgraOBP9, AgraOBP10, and AgraOBP11, which were up-regulated by both stimuli in female antennae. Another was AgraOBP14, AgraOBP16, AgraOBP18, AgraOBP21, and AgraOBP22, which were down-regulated by both semiochemicals in females. A third group was AgraOBP2, AgraOBP7, AgraOBP20, and AgraGOBP1, which were down-regulated in male antennae to both stimuli. None of the AgraOBPs responded similarly to both semiochemicals in both females and males. If these were all regulated to enhance ligand-specific detection, it is unclear how they would respond to both groups of compounds and why so many OBPs would respond to both groups. The pheromone compounds and the plant volatiles do not share structural similarities. The four pheromone compounds are two circular terpene alcohols and two circular terpene aldehydes and the eight plant volatiles are four linear 6-carbon compounds, two linear terpenes, an alkene terpene, and a sesquiterpene. Alternatively, the regulation of these OBPs could be a coordinated response to prime the olfactory system to detect other environmental semiochemicals.

### Systemic response: a new view of OBP expression

In previous works, constitutive levels of expression of OBPs in female and male antennae have been directly used to suggest that some OBPs respond to host and/or sex-attractants without actually testing the effect of such stimuli on their expression [*e.g*.^10,11^]. Such interpretations require the assumption that exposure to semiochemical stimuli does not affect the expression of OBPs. Although the constitutive expression patterns are suggestive of specific functional responses, it is critical to use semiochemical stimulus assays to test these ideas. This assumption that AgraOBP expression is not affected by exposure to semiochemicals was clearly rejected for boll weevil because it generated systemic responses in the expression of AgraOBPs that differed for the two stimuli and for the two sexes. Our recent paper^79^ on brown marmorated stink bug also showed that stimulation by either alarm pheromone or aggregation pheromone caused a systemic change in expression of multiple OBPs.

The observed systemic response might serve to enhance detection of the semiochemical that caused the response and/or act as a cue to prime the olfactory system to detect other environmental chemicals. If semiochemical stimulation changed expression of OBPs to enhance detection of the semiochemical, then it seems reasonable to infer that AgraOBPs should be up-regulated by one of the stimuli, pheromone or plant volatiles, but not both. Indeed, as many as four OBPs might be up-regulated to enhance detection of the four pheromone compounds and as many as eight OBPs might be up-regulated to enhance detection of the plant volatiles. Thus, some of the up-regulated OBPs might serve this purpose. Alternatively, if semiochemical stimulation changed expression to prime the olfactory system to detect other environmental chemicals, some AgraOBPs might be up-regulated by both semiochemicals. Females up-regulated AgraOBP5, AgraOBP9, AgraOBP10 and AgraOBP11 in response to both pheromone and plant volatiles consistent with the priming possibility. As males did not up-regulate any AgraOBPs, the male response does not distinguish these possibilities.

Considering that enhanced detection occurs by up-regulation or turning on the expression of one or more OBPs, enhanced detection is not necessarily mutually exclusive with olfactory priming. Females exclusively up-regulated or turned on seven AgraOBPs when stimulated only by pheromone (AgraOBP3, AgraOBP4, AgraOBP6, AgraOBP8, AgraOBP12, AgraOBP20, and AgraOBP23). So, any of these AgraOBPs could be used to enhance detection of at least one of the four pheromone components. In the same way, females exclusively up-regulated AgraGOBP1 only when stimulated by plant volatiles, and therefore it could be used to enhance detection of at least one of the eight plant volatiles.

These results are consistent with the known chemical ecology of female boll weevils, which use pheromone^14–16^ enhanced by green leaf volatiles^18^ to locate males and host plants. Males, in contrast, did not up-regulate or turn on any AgraOBP in response to these pheromones or plant volatiles, which is consistent with the known chemical ecology of male boll weevils regarding pheromone attraction. However, it would be expected that males would up-regulate or turn on some AgraOBPs in response to some plant volatiles to locate host plants. We used a mixture of eight compounds that when tested individually provoked boll weevil attraction behaviour and/or EAG and/or single-neuron responses^16,18–21,44^. The combination of all eight plant volatiles together might have provided conflicting signals to males. The four 6-carbon compounds are general green leaf volatiles^18,80^, which provide non-specific plant cues. The other four are known to be released by cotton during the flowering and fruiting period^24,31–34^, but are released at much higher levels when attacked by leaf-chewing herbivores^23,26–30^. Alternatively, green leaf volatiles might lead to enhanced detection of cotton-specific volatiles by down-regulating unrelated AgraOBPs, or the other four plant volatiles might signal plants that should be avoided. All of these are variations on the priming possibility, as none involve enhanced detection of the compounds we used in this study.

Thus, it is possible that the systemic changes in expression of AgraOBPs in response to pheromone or plant volatiles enhance detectability of these stimuli and prime the olfactory system to detect other environmental chemicals. As more information about the function of these AgraOBP transcripts accrues, their function will become clarified. Whether the systemic response was to enhance detection or prime the olfactory system, AgraOBPs that were down-regulated to both pheromone and plant volatile stimuli suggest that they might be involved in olfactory processes other than those associated with the pheromone and plant volatile stimuli used here.

### Data availability

The datasets generated during and/or analyzed during the current study are available from the corresponding author on reasonable request.

## Electronic supplementary material

Supplementary Information 1

Supplementary Information 2
