# Supplementary material for: Systemic and sex-biased regulation of OBP expression under semiochemical stimuli
Source: Sci Rep. 2018 Apr 16;8:6035. doi: 10.1038/s41598-018-24297-z (PMC5902564; doi:10.1038/s41598-018-24297-z)
Supplement: Supplementary file 1 — Supplementary Information 1 [file 41598_2018_24297_MOESM1_ESM.pdf]

Supplementary Information 1

Systemic and sex-biased regulation of OBP expression under semiochemical stimuli

Débora Pires Paula, Roberto Coiti Togawa, Marcos Mota do Carmo Costa, Priscila Grynberg, Natália Martins Florêncio, David Alan Andow

Methods

In silico characterization of the OBP transcripts

The full-length putative OBP transcripts were submitted to *in silico* analyses to predict the deduced amino acid sequences (http://web.expasy.org/translate/), the pI and monoisotopic molecular weight (MW) (COMPUTE PI/MW, http://web.expasy.org/compute_pi/), the presence of the signal peptide (SIGNALP 4.1, http://etal.cbs.dtu.dk/services/SignalP/) [S1], and the location of the PBP/GOBP domain (http://www.ebi.ac.uk/interpro/) [S2]. Their predicted tertiary structures were generated using the online program MODELLER at Protein Structure Prediction Server version 3.0 (PS2 3.0) [S3]. Several templates were selected by sequence identity (Table S1 in this Supporting Information). The overall amino acid identity between target and template varied from 60.49% to 88.15%. Models were displayed using the VMD software [S4].

Table S1. 3D molecular modeling prediction for full-length putative *Anthonomus grandis* OBPs according to PS2 server.

Sequence	Template	Aligned (%)	Identity (%)	Bit-score	E-value
AgraOBP2	no significant template				

AgraOBP3	1c3yA	81.2	17.59	211	1.30E-05
AgraOBP4	no significant template				
AgraOBP5	2erbA	73.83	26.13	193.7	0.00012
AgraOBP6	2erb	88.15	29.75	272.4	4.80E-09
AgraOBP7	1c3yA	76.76	26.61	212	1.10E-05
AgraOBP8	1dqeA	83.56	22.05	162.8	0.0061
AgraOBP9	1c3yA	66.19	26.09	189.2	0.00021
AgraOBP10	1c3yA	60.49	22.45	179.8	0.00069
AgraOBP11	1c3yA	85.45	32.63	215.2	7.40E-06
AgraOBP12	1c3yA	75.61	33.33	180.3	0.00065
AgraOBP13	1c3yA	80.88	25.45	208.1	1.80E-05
AgraOBP14	no significant template				
AgraOBP15	no significant template				
AgraOBP16	2erbA	87.31	26.05	189.9	0.00019
AgraOBP18	no significant template				
AgraOBP19	no significant template				
AgraOBP21	1c3yA	75.59	14.85	1751.1	0.0013
AgraOBP23	2erbA	86.23	23.53	191.4	0.00016

We also analyzed the similarity among the deduced amino acid sequences of the putative full-length OBPs identified in the boll weevil using MAFFT 7.017 [S5] with the algorithm auto, scoring matrix BLOSUM62, gap opening penalty 1.53 and offset value of 0.123, in Geneious 7.0.5 [S6]. This analysis enabled the identification of the conserved Cys motif-pattern. The similarity of the AgraOBPs was also compared with 256 coleopteran OBPs from 19 species (including 112 curculionid OBPs from nine species) available in DDBJ/EMBL/GenBank.

Chemical sources of the semiochemicals

Boll weevil aggregation pheromone (Grandlure) and plant volatile organic compounds were tested separately. Grandlure (GL) components were obtained from

Bedoukian Research (Danbury, CT, www.bedoukian.com). Grandlure component I (GL-I) was (+)-(E)-1-methyl-2-isopropenyl-cyclobutane ethanol (95.7% purity by gas liquid chromatography-GLC), Grandlure component II (GL-II) was (Z)-3,3-dimethyl-$\Delta^{1,\beta}$-cyclohexane ethanol (99.2% purity by GLC), and Grandlure component III/IV (GL-III/IV) was a mixture of 48.6% (Z)-3,3-dimethyl-$\Delta^{1,\alpha}$-cyclohexane acetaldehyde and 47.9% (E)-3,3-dimethyl-$\Delta^{1,\alpha}$-cyclohexane acetaldehyde. Optimal mixtures of these components were studied by Hardee et al. [S7], who found that a mixture of 50:40:10 for GL-I: GL-II: GL-III/IV was repeatedly more attractive than other blends (such as 22.5:17.5:60 by Tumlinson et al. [S8]; 30:40:30 by Dickens [S9]; 38:28:33 by Showler et al. [S10]; 35:43:22 in commercial Grandlure by Magalhães et al. [S11]). We mixed the Grandlure components in this ratio in *n*-hexane (1 µL Grandlure per 25 µL of solution). This mixture was used within 48 h, and stored at -5°C when not in use.

The attractiveness of plant volatile organic compounds (PVOC) to boll weevils has been studied by many authors with some inconsistent results [S9,S11-S22]. Based on this literature, we used hexanal (98% purity, Sigma-Aldrich, St. Louis MO), (E)-2-hexen-1-ol (96% purity, Acros Organics, New Jersey, www.acros.com), (E)-2-hexenal (99% purity, Acros Organics), 1-hexanol (98% purity, Merck KGaA via EMD Millipore, Billerica, MA), linalool (97% purity, Spectrum Chemical, Gardena, CA), (E)-β-caryophyllene (90% purity, Tokyo Chemical Industry, Tokyo), α-pinene (98% purity, Sigma-Aldrich, St. Louis MO), and (E)-β-ocimene (90% purity, mixture of isomers, Sigma-Aldrich, St. Louis MO). These eight chemicals were known to stimulate an electroantennogram and/or behavioral response in boll weevils [S11-S15], and were combined in *n*-hexane (1 µL of each compound per 25 µL of solution). Dickens [S15] showed that boll weevil electroantennogram (EAG) responses saturated at this concentration. The PVOC mixture was used within 48 h, and stored at -5°C when not in use.

References

S1. Petersen, T. N., Brunak, S., von Heijne, G. & Nielsen, H. SignalP 4.0: discriminating signal peptides from transmembrane regions. *Nat. Methods* **8** (10), 785 (2011).

S2. Finn, R. D. *et al*. InterPro in 2017 - beyond protein family and domain annotations. *Nucleic Acids Res*. **45**, D190 (2017).

S3. Huang, T.-T. *et al.* (PS)2: protein structure prediction server version 3.0. *Nucleic Acids Res*. **43**, W338 (2015).

S4. Humphrey, W., Dalke, A. & Schulten, K. "VMD - Visual Molecular Dynamics". *J. Mol. Graph*. **14**, 33 (1996).

S5. Katoh, K., Misawa, K., Kuma, K. I. & Miyata, T. MAFFT: a novel method for rapid multiple sequence alignment based on fast Fourier transform. *Nucleic Acids Res.* **30** (14), 3059 (2002).

S6. Kearse, M. *et al*. Geneious Basic: an integrated and extendable desktop software platform for the organization and analysis of sequence data. *Bioinformatics* **28** (12), 1647 (2012).

S7. Hardee, D. D., McKibben, G. H., Rummel, D. R., Huddleston, P. M. & Coppedge, J. R. Response of boll weevils to component ratios and doses of the pheromone, Grandlure. *Environ. Entomol*. **3** (1), 135 (1974).

S8. Tumlinson, J. H., Hardee, D. D., Gueldner, R. C., Thompson, A. C. & Hedin, P. A. Sex pheromones produced by male boll weevil: isolation, identification, and synthesis. *Science* **166** (3908), 1010 (1969).

S9. Dickens, J. C. Orientation of boll weevil, *Anthonomus grandis* Boh. (Coleoptera: Curculionidae), to pheromone and volatile host compound in the laboratory. *J. Chem. Ecol*. **12**, 91 (1986).

S10. Showler, A. T., Robacker, D. & Salgado, E. Grandlure dosage and attraction of boll weevils (Coleoptera: Curculionidae). *J. Econ. Entomol*. **99**, 1675 (2006).

S11. Magalhães, D. M. *et al*. Semiochemicals from herbivory induced cotton plants enhance the foraging behavior of the cotton boll weevil, *Anthonomus grandis*. *J. Chem. Ecol*. **38**, 1528 (2012).

S12. Dickens, J. C. Olfaction in the boll weevil, *Anthonomus grandis* Boh. (Coleoptera: Curculionidae): Electroantennogram studies. *J. Chem. Ecol*. **10**, 1759 (1984).

S13. Dickens, J. C. Green leaf volatiles enhance aggregation pheromone of boll weevil, *Anthonomus grandis*. *Entomol. Exp. Appl*. **52**, 191 (1989).

S14. Gueldner, R. C., Thompson, A. C., Hardee, D. D. & Hedin, P. A. Constituents of cotton bud. XIX. Attractancy to the boll weevil of the terpenoids and related plant constituents. *J. Econ. Entomol*. **63**, 1819 (1970).

S15. Dickens, J. C. Specialized receptor neurons for pheromones and host plant odors in the boll weevil, *Anthonomus grandis* Boh. (Coleoptera: Curculionidae). *Chem. Senses*. **15**, 311 (1990).

S16. Minyard, J. P. *et al*. Constituents of the cotton bud, compounds attractive to the boll weevil. *J. Agr. Food Chem*. **17**, 1093 (1969).

S17. Magalhães, D. M. et al. Influence of two acyclic homoterpenes (tetranorterpenes) on the foraging behavior of *Anthonomus grandis* Boh. *J. Chem. Ecol*. **42**, 305 (2016).

S18. McCall, P. J., Turlings, T. C. J., Loughrin, J., Proveaux, A. T. & Tumlinson, J. H. Herbivore-induced volatile emissions from cotton (*Gossypium hirsutum* L.) seedlings. *J. Chem. Ecol.* **20**, 3039 (1994).

S19. Loughrin, J. H., Manukian, A., Heath, R. R. & Tumlinson, J. H. Volatiles emitted by different cotton varieties damaged by feeding beet armyworm larvae. *J. Chem. Ecol*. **21**, 1217 (1995).

S20. Paré, P. W. & Tumlinson, J. H. Cotton volatiles synthesized and released distal to the site of insect damage. *Phytochemistry* **47**, 521 (1998).

S21. Rodriguez-Saona, C., Crafts-Brandner, S. J. C. & Caňas, L. A. Volatile emissions triggered by multiple herbivore damage: beet armyworm and whitefly feeding on cotton plants. *J. Chem. Ecol*. **29**, 2539 (2003).

S22. Moraes, M. C., Laumann, R. A., Aquino, M. F. S., Paula, D. P. & Borges, M. Effect of *Bt* genetic engineering on indirect defense in cotton via a tritrophic interaction. *Transgenic Res*. **20**, 99 (2011).
