# Supplementary material for: Systemic and sex-biased regulation of OBP expression under semiochemical stimuli
Source: Sci Rep. 2018 Apr 16;8:6035. doi: 10.1038/s41598-018-24297-z (PMC5902564; doi:10.1038/s41598-018-24297-z)

Supplementary Information 2

Systemic and sex-biased regulation of OBP expression under semiochemical stimuli

Débora Pires Paula, Roberto Coiti Togawa, Marcos Mota do Carmo Costa, Priscila Grynberg, Natália Martins Florêncio, David Alan Andow

Table S1. Primer pairs (5'>3') designed for expression analysis of OBPs in boll weevil by qPCR.

Agra_Actin1_F	AGGCACAGACAAGAAGAGGC
Agra_Actin1_R	CAGGGTGTTCTTCAGGTGCT
Agra_αTubulin_F	AGAGGAGACGTCGTACCCAA
Agra_αTubulin_R	CTCCGCCCACTACAACTGTT
AgraEF1_F	TGGTAACTGGTGCTGCTCAA
AgraEF1_R	AGCAACCAAGGACAGCAGAA
Agra_GAPDH_F	GTCCATGCGATCACAAACGA
Agra_GAPDH_R	CCCAATGCCTTCGATGCAAC
AgraGOBP_F	CTTCTGCAATGCGTTTACCGT
AgraGOBP_R	ACAGCCTGTAATGTTGCCAGA
AgraOBP1_F	CAAATGCCAAGCTGACCCTG
AgraOBP1_R	TTGTTGATTGCGCCATCAGC
AgraOBP2_F	TGAACCCAGAAGGCATCACC
AgraOBP2_R	AATGGGTTTTTCGTTGGCGG
AgraOBP3_F	GCATGATGGTGGACCTTGGA
AgraOBP3_R	GTTTCCATGCTGGTTGGAGC
AgraOBP4_F	AAAACCAAGAGGGGGGCTGTC
AgraOBP4_R	GAAAGCGGTCTCCTGAGGAC
AgraOBP5_F	AGGAAAAACTACCAGCCGGG
AgraOBP5_R	CAGGGTGGGTATGCTCGAAG
AgraOBP6_F	TCACAACCATCGAGGCCAAA
AgraOBP6_R	ATCGGTCTCTTGCAAACCCC
AgraOBP7_F	ACCGACGAAGCTAAGCAACT
AgraOBP7_R	TTGGCACTTTCTTCGGGAGT
AgraOBP8_F	GCAAACCTCCGGTAACCCAT
AgraOBP8_R	GGATCGTCCGCCTTAGCTTT
AgraOBP9_F	AGTGGTGTCTGCCAAGAAGG
AgraOBP9_R	TCCACTTTGGTTGCCTCGTT
AgraOBP10_F	GGACACGTCGAGAAAGACCA
AgraOBP10_R	ATGATGATGATGGGGTCCGC
AgraOBP11_F	CGATGCCGGAGATATCCAGG
AgraOBP11_R	AAGCCGTATCCTCGTTGGTG
AgraOBP12_F	GACGGGCAATTTTCCAGACG
AgraOBP12_R	GCTTTGGCTTCATCACCGAG
AgraOBP13_F	ATAGCTTGAACACCGCCGAA
AgraOBP13_R	TTTCCTCTGAGCTGCCCTTG
AgraOBP14_F	GTCGTCTCCCAAGTGCTGAA
AgraOBP14_R	GAATCGTTTACCGTCGTGCG
AgraOBP15_F	GTGGGCGTAGACAAGGATGA
AgraOBP15_R	ATCGTCTTTCAACCAGCCGT
AgraOBP16_F	CGTGACAGACACCGGAGTAG
AgraOBP16_R	TGGAAGCCGATTTTCGTGGA
AgraOBP17_F	ACGTCGAGGGAGTGAAAACC
AgraOBP17_R	GCTGCTCTCGTTCTCCTTGT
AgraOBP18_F	AGTGCCGCTAATAACGCTTT
AgraOBP18_R	GGAACCGTCCGAATTTTGCC
AgraOBP19_F	TAAGCACTCCTCCTCCACCT
AgraOBP19_R	TTGGGCTCAGGGATTTCTGT
AgraOBP20_F	GCTGCTTAGGACCCAACACT
AgraOBP20_R	TCAGTTTCGGTTCGTCAGCT
AgraOBP21_F	CCACGGAAATGAAGCAAATCGT
AgraOBP21_R	CAGTCGCTTCAGGACTACCC
AgraOBP22_F	TCGATGAGCACAATATGGCT
AgraOBP22_R	ACAGACCGTGTTCGTGCTTA
AgraOBP23_F	CTGTCCGTTATTGTTGCCGC
AgraOBP23_R	CGTCGGTGTATTCTCCGGTC

Table S2. Statistics of the completeness of the boll weevil transcriptome based on the Core Eucaryotic Genes Mapping Approach - CEGMA.

	#Prots	%Completeness	-	#Total	Average	%Ortho
**Complete**	**118**	**47.58**	**-**	**297**	**2.52**	**79.66**
Group 1	22	33.33	-	61	2.77	81.82
Group 2	23	41.07	-	62	2.70	82.61
Group 3	33	54.10	-	86	2.61	78.79
Group 4	40	61.54	-	88	2.20	77.50
**Partial**	**168**	**67.74**	**-**	**462**	**2.75**	**82.14**
Group 1	35	53.03	-	101	2.89	88.57
Group 2	36	64.29	-	98	2.72	80.56
Group 3	47	77.05	-	134	2.85	78.72
Group 4	50	76.92	-	129	2.58	82.00

These results are based on the set of genes selected by Parra G, Bradnam K, Korf I. (2007) CEGMA: a pipeline to accurately annotate core genes in eukaryotic genomes. Bioinformatics, 23: 1061-1067.

Key:
Prots = number of 248 ultra-conserved CEGs present in genome
%Completeness = percentage of 248 ultra-conserved CEGs present
Total = total number of CEGs present including putative orthologs
Average = average number of orthologs per CEG
%Ortho = percentage of detected CEGS that have more than 1 ortholog

Table S3. Putative OBP identified in Coleoptera. *Anthonomus grandis* OBPs identified in this work are not included in this table.

Coleopteran species	OBP	References
Aethina tumida	14	GenBank
Agrilus planipennis	10	Mamidala et al. (2013)
Ambrostoma quadriimpressum	16	Wang et al. (2016)
Anomala corpulenta	21	Li et al. (2015)
Anomala cuprea	2	Nikonov et al. (2002)
Anomala octiescostata	2	Nikonov et al. (2002)
Anomala osakana	1	Wojtasek et al. (1999)
Anomala rufocuprea	4	GenBank
Anomala schonfeldti	5	GenBank
Anoplophora glabripennis	44	Hu et al. (2016)
Batocera horsfieldi	12	Li et al. (2014)
Colaphellus bowringi	26	Li et al. (2015a)
Cryptolaemus montrouzieri	2	Chang et al. (2016)
Cybister japonicus	2	Song et al. (2016)
Cyrtotrachelus buqueti	36	Yang et al. (2017)
Dastarcus helophoroides	33	Li et al. (2015b)
Dendroctonus armandi	11	GenBank
Dendroctonus ponderosae	56	Andersson et al. (2013)
Dendroctonus valens	21	Gu et al. (2015)
Diabrotica virgifera virgifera	29	Xu et al. (2009)
Diaprepes abbreviatus	3	Xu et al. (2009)
Exomala orientalis	2	Peng et al. (2001)
Galeruca daurica	29	Li et al. (2017)
Harmonia axyridis	1	Vandermoten et al. (2011)
Heptophylla picea	1	Deyu and Leal (2002)
Holotrichia oblita	4	Deng et al. (2012); Wang et al. (2013)
Holotrichia parallela	27	Ju et al. (2014)
Hylamorpha elegans	6	González-González et al. (2016)
Hypothenemus hampei	2	Xu et al. (2009)
Ips acuminatus	1	GenBank
Ips typographus	15	Andersson et al. (2013)
Leptinotarsa decemlineata	26	Liu et al. (2015a) and Xu et al. (2009)
Lissorhoptrus oryzophilus	10	Yuan et al. (2016)
Monochamus alternatus	52	Wang et al. (2014); Gao and Wang (2015)
Nicrophorus vespilloides	25	GenBank
Phyllopertha diversa	4	Wojtasek et al. (1999)
Phyllotreta striolata	32	Wu et al. (2016)
Popillia japonica	1	Wojtasek et al. (1999)
Pyrrhalta aenescens	31	Zhang et al. (2016)
Pyrrhalta maculicollis	36	Zhang et al. (2016)
Rhynchophorus ferrugineus	14	Yan et al. (2016)
Rhynchophorus palmarum	4	Meillour et al. (2004)
Rhyzopertha dominica	16	Diakite et al. (2016)
Tenebrio molitor	19	Liu et al. (2015b)
Tomicus yunnanensis	11	Zhu et al. (2012)
Tribolium castaneum	49	Richards et al. (2008)
**Total**	768	
**Average**	16.7	
**STDEV**	15.3	

Figure S1. Distribution of *p-values*. Tests of difference between semiochemical treatment and control for antennae and legs of females and males for all OBP transcripts and all treatments.

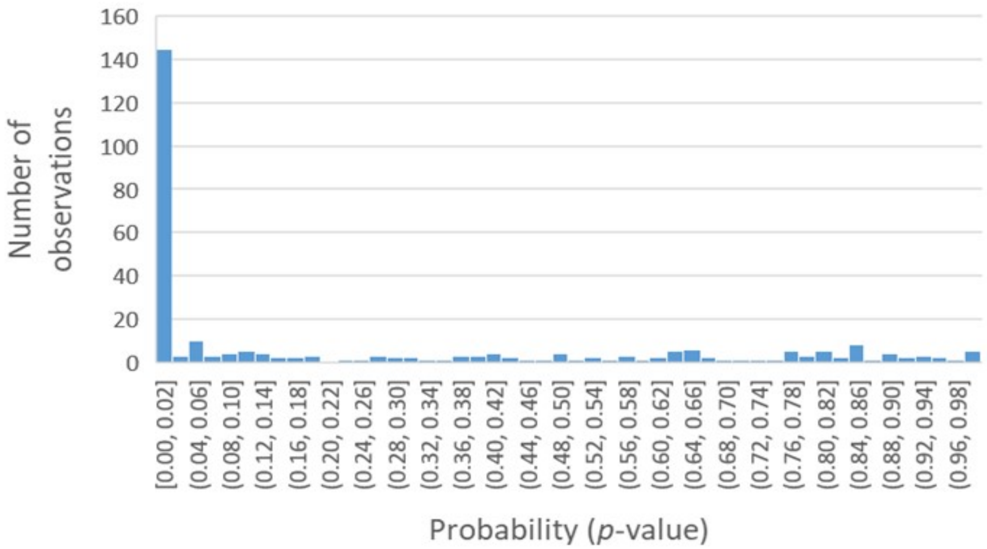

Figure S2. Three dimensional models for *Anthonomus grandis* full-length putative:
A) AgraOBPs; B) AgraOBP3 showing six numbered segments in alpha-helices and the hydrophobic cavity lined with several hydrophobic amino acids between alpha-helices 4 and 6 (Tyr72, Ile80, Thr81, Ile84, Val101, Phe120, Phe123), which are highlighted in the model; C) Zoom in on these hydrophobic amino acids. Secondary structure is colored, with alpha-helice in magenta, loops and coils in white and blue and beta-strands in yellow.

A.

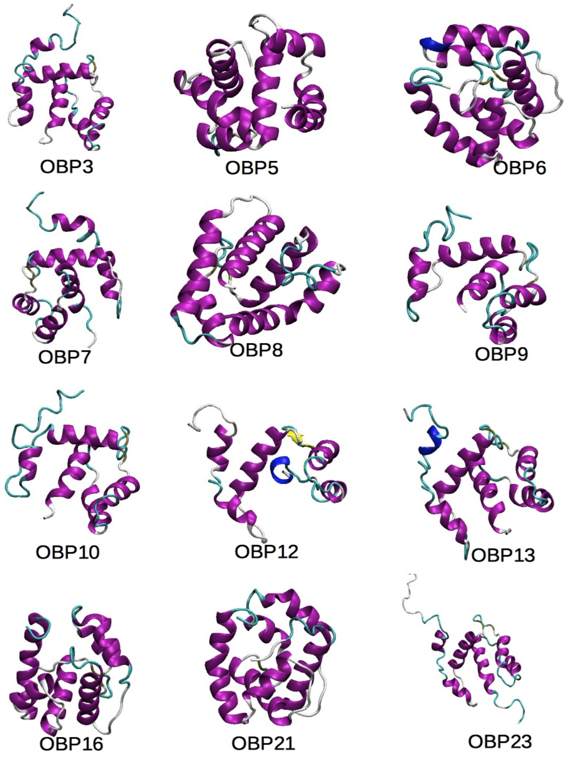

B.

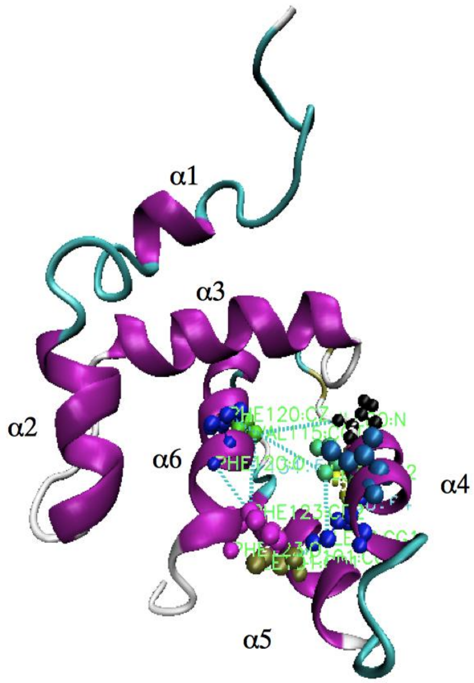

C.

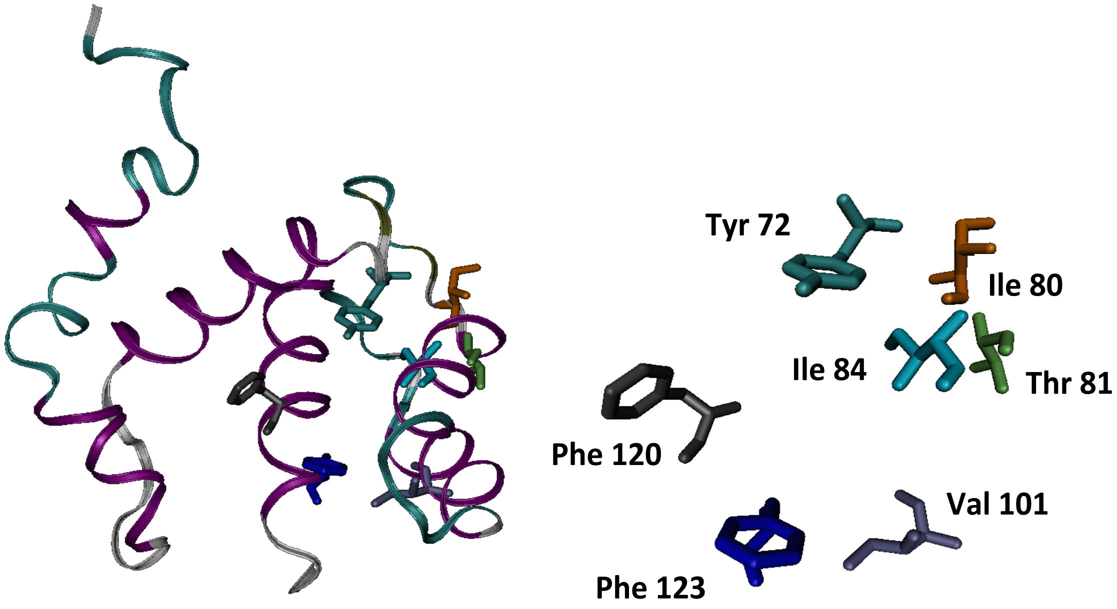

Fig. S3. Expression of OBP transcripts in stimulated boll weevils. Expression is in log10 relative fluorescence units (N0) corrected for plate effects and normalized to two reference genes with standard errors and sample size. GFA: Grandlure female antennae; GMA: Grandlure male antennae; PFA: plant volatiles female antennae; PMA: plant volatiles male antennae. Statistical difference between stimulus and control is indicated with * = p<0.01; ** = p<0.001; *** = p<0.0001. OFF: turned-off; NE: not estimated; STAY OFF: remained unexpressed.

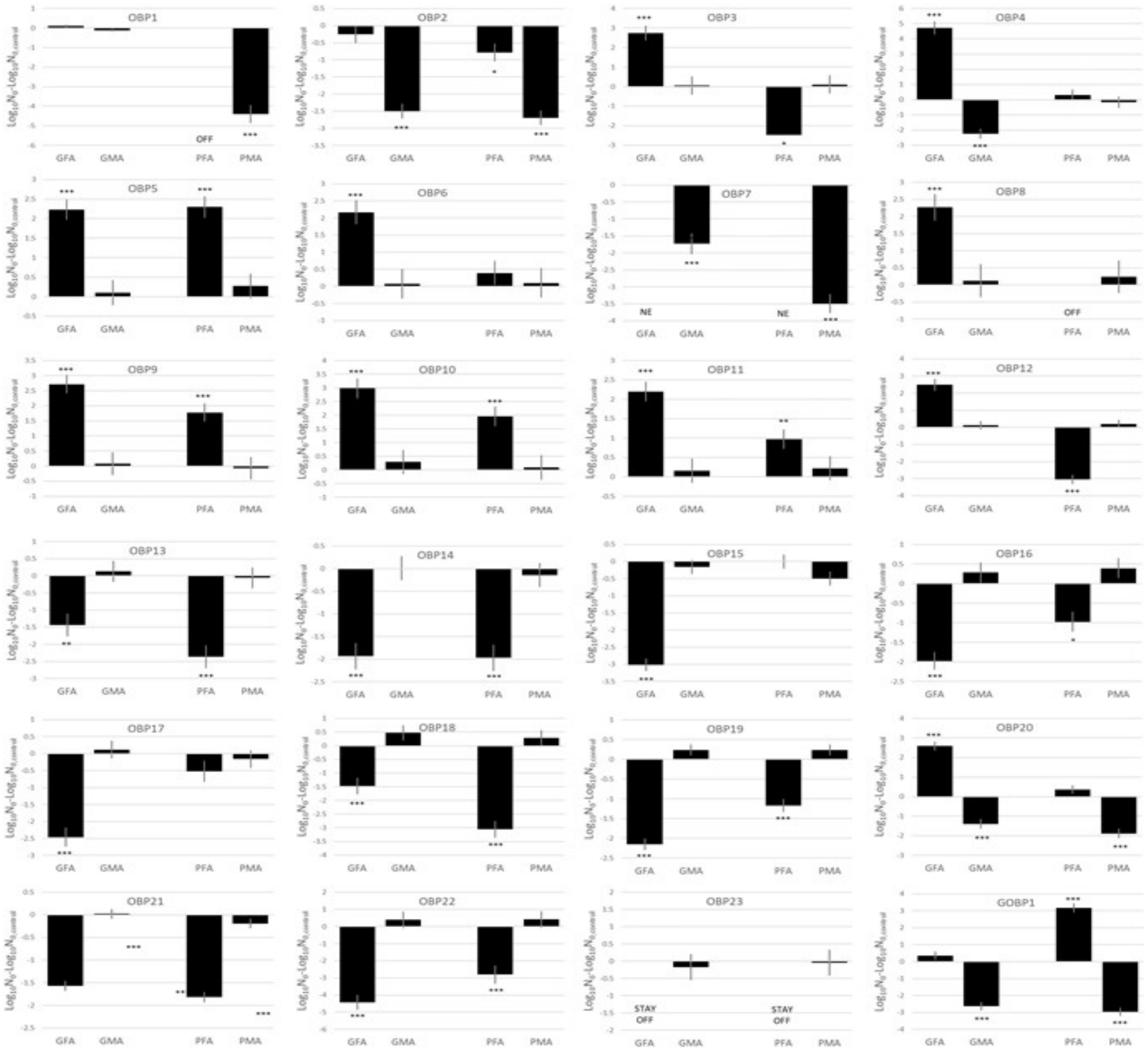

Supplement: Supplementary file 2 — Supplementary Information 2 [file 41598_2018_24297_MOESM2_ESM.pdf]
